# Adaptive-mixture-categorization (AMC)-based g-computation and its application to trace element mixtures and bladder cancer risk

**DOI:** 10.1038/s41598-022-21747-7

**Published:** 2022-10-25

**Authors:** Siting Li, Margaret R. Karagas, Brian P. Jackson, Michael N. Passarelli, Jiang Gui

**Affiliations:** 1 Quantitative Biomedical Sciences Program, Geisel School of Medicine at Dartmouth, Hanover, NH USA; 2 Department of Epidemiology, Geisel School of Medicine at Dartmouth, Hanover, NH USA; 3 Trace Element Analysis Laboratory, Department of Earth Sciences, Dartmouth College, Hanover, NH USA; 4 Department of Biomedical Data Science, Geisel School of Medicine at Dartmouth, Hanover, NH USA

**Keywords:** Computational biology and bioinformatics, Bladder

## Abstract

Several new statistical methods have been developed to identify the overall impact of an exposure mixture on health outcomes. Weighted quantile sum (WQS) regression assigns the joint mixture effect weights to indicate the overall association of multiple exposures, and quantile-based g-computation is a generalized version of WQS without the restriction of directional homogeneity. This paper proposes an adaptive-mixture-categorization (AMC)-based g-computation approach that combines g-computation with an optimal exposure categorization search using the F statistic. AMC-based g-computation reduces variance within each category and retains the variance between categories to build more powerful predictors. In a simulation study, the performance of association analysis was improved using categorizing by AMC compared with quantiles. We applied this method to assess the association between a mixture of 12 trace element concentrations measured from toenails and the risk of non-muscle invasive bladder cancer. Our findings suggested that medium-level (116.7–145.5 μg/g) vs. low-level (39.5–116.2 μg/g) of toenail zinc had a statistically significant positive association with bladder cancer risk.

## Introduction

Mixture analysis is commonly used in environmental epidemiology. Several studies have analyzed the association between a health outcome and a specific mixture, including the mixture of air pollutants (e.g. PM_2.5_, NO_2_)^1,2^, the mixture of persistent organic pollutants (POPs)^3,4^, and the mixture of per- and polyfluoroalkyl substances (PFAS)^5,6^. Since humans are exposed to multifarious mixtures of chemical elements via air, soil, food, and water, the joint effect of a mixture of chemical elements may better reflect the “real world” scenario. Analyses accounting for diverse mixtures of chemical elements could lead to a more comprehensive risk assessment than single-element approaches^7^.

Weighted quantile sum (WQS) regression^8^ is a popular method for assessing the association between mixture of exposures and health outcome. WQS defined the index of the overall mixture as a weighted linear combination of all exposures. Then it applies a weighted index model^9^ to estimate the mixture effect. A limitation of WQS regression is that it assumes all associations are in the same direction (i.e., exposures in a mixture are either all positively or all negatively associated with the outcome). To overcome this limitation, Keil et al. developed an alternative method called quantile-based g-computation for mixture analysis that eliminates the restriction of directional homogeneity^10^. Like WQS, quantile-based g-computation first transforms the exposures into quantiles and uses generalized linear model to estimate the exposure effects in reference to the lowest quantile. If all the exposure effects are either all positive or all negative, then quantile-based g-computation is asymptotically equivalent to WQS. Otherwise, it redefines the weights to be positive and negative weights. In the way, both directions are taken into consideration. In contrast to WQS which estimates weights that sum to one within each bootstrapping, quantile-based g-computation permits positive weights and negative weights to both sum to one.

Both WQS regression and quantile-based g-computation transform the continuous mixture variables into quantiles (default setting) which is a common practice in epidemiology to account for a pre-specified nonlinear relationships between the exposures and outcome^11^. The categorization can reduce the impact of outliers and make the regression analysis more robust compared to continuous exposures and can enhance the interpretability of regression coefficients. However, this approach is not flexible because it uses fixed percentiles of the data as the cutoffs, which may not be the optimal categorization minimizing the variance within each category. In real data analysis, it is not always apparent without examining the distribution of each exposure to determine the most appropriate number of categories to use. In this paper, we propose the adaptive-mixture-categorization (AMC)-based g-computation approach, which utilizes a general F-statistic and a linear search strategy to optimally categorize the exposures based on its variation. By maximizing the F-statistic during the categorization, AMC minimizes the within-category variation and maximizes the between-category variation to improve the accuracy for variable selection. G-computation is then applied to the AMC-categorized exposures to estimate the mixture effect. This AMC-based g-computation approach, which does not fix cutoff percentiles nor the number of categories, is more flexible than quantile-based g-computation.

In real data analysis, we applied our approach to toenail trace element mixture data from the New Hampshire Bladder Cancer Study. In the United States, bladder cancer is the fourth most common malignancies among men^12,13^. Worldwide, there are approximately 430,000 incidents of bladder cancer and 165,000 deaths from bladder cancer per year^14^. Toxic element exposure is a well-known risk factor for bladder cancer, but most studies consider one element at a time^15,16^. For example, an age- and sex-matched case–control study from New England found low-to-moderate levels of arsenic from drinking water was associated with higher bladder cancer risk^15^. Few previous studies have evaluated the association between trace element mixtures and bladder cancer. This study was designed to bridge this gap using a novel mixture method.

## Results

### Comparisons of methods on simulated datasets

**Figure 1:**
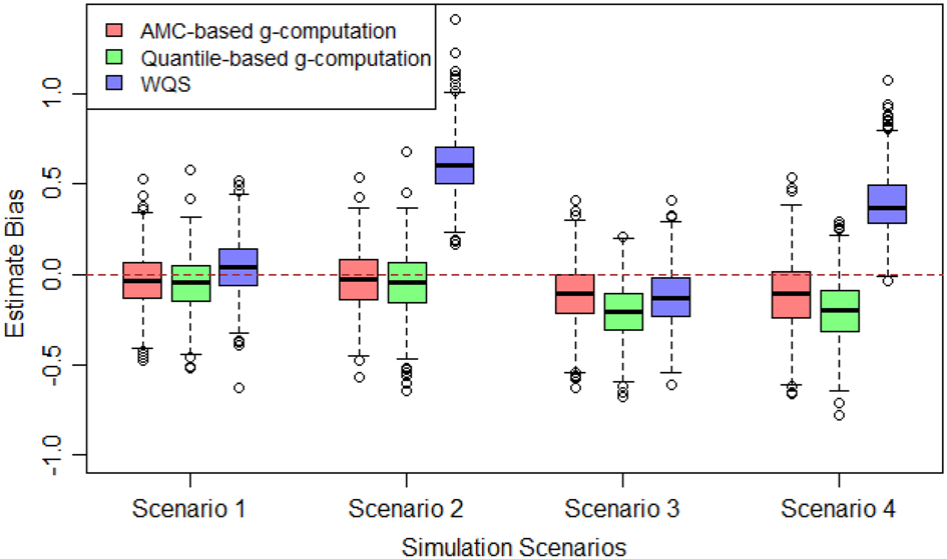
The estimate bias of the mixture effect. $Bias = \psi_{estimate} - \psi_{true}$. *ψ* was the effect of the mixture. Bias was calculated across 1000 simulations. The sample size of each exposure was 200. For more details of the meaning for parameters and simulation design, please refer to the “Methods and materials”. Scenario 1: Equal proportions: ${\upalpha }_{j0} = {\upalpha }_{j1} = {\upalpha }_{j2} = {\upalpha }_{j3} = 0.25$; Effects are both positive: $\beta_{1} = \beta_{2} = 0.5$; $\psi_{true} = 1$ Scenario 2: Equal proportions: ${\upalpha }_{j0} = {\upalpha }_{j1} = {\upalpha }_{j2} = {\upalpha }_{j3} = 0.25$; Effects are positive and negative:${ }\beta_{1} = 1.5,\;\beta_{2} = - 0.5$; $\psi_{true} = 1$ Scenario 3: Unequal proportions: ${\upalpha }_{j0} = 0.4,\;{\upalpha }_{j1} = 0.3,\;{\upalpha }_{j2} = 0.2,\;{\upalpha }_{j3} = 0.1$; Effects are both positive: $\beta_{1} = \beta_{2} = 0.5$; $\psi_{true} = 1$ Scenario 4: Unequal proportions: ${\upalpha }_{j0} = 0.4,\;{\upalpha }_{j1} = 0.3,\;{\upalpha }_{j2} = 0.2,\;{\upalpha }_{j3} = 0.1$; Effects are positive and negative: ${ }\beta_{1} = 1.5,\;\beta_{2} = - 0.5$; $\psi_{true} = 1$

The first simulation compared the performance of AMC-based g-computation, quantile-based g-computation and WQS by calculating the estimation bias of the mixture effect. We simulated each exposure from a mixture of four normal distributions, with both equal (scenarios 1 and 2) and unequal proportions (scenarios 3 and 4) of distribution. As shown in Fig. 1, for scenario 1, WQS, quantile-based g-computation and AMC-based g-computation achieved similar absolute value of average bias across the 1000 simulations. For scenario 2, AMC-based g-computation and quantile-based g-computation both had good effect estimates with a very small average bias, while WQS generated a large positive average bias of the joint mixture effect because the directional homogeneity assumption was not valid. For scenarios 3 and 4, when the distribution proportions were unequal, AMC-based g-computation achieved the smallest average bias among all three methods. In scenario 4, WQS generated a large bias again because the exposure effects were not in the same direction. Relative to using fixed quantiles, AMC improved the performance of the joint effect estimate by categorizing more exposures into the correct hidden state rather than categorizing them by fixed percentiles. Overall, quantile-based g-computation outperformed WQS because of its capability to account for bidirectional effect estimates, but AMC-based g-computation still achieved the smallest average bias in all cases.

The second simulation evaluated the variable-selection performance by calculating the True Positive Rate (TPR) of identifying the correct causal variables. We assessed both monotonic effects (Table 1) and non-monotonic effects (Table 2). Each simulation was repeated 1000 times to calculate the average TPR. The average TPRs of AMC-based g-computation were the highest among all scenarios. They were 2–3% higher than quantile-based g-computation under monotonic effects, showing that categorization by AMC could improve the variable-selection performance. Quantile-based g-computation performed satisfactorily by using ordinal variables that maintained the order of the continuous raw data. WQS assigned all exposures with positive weights because the joint mixture effect was positive, which led to the lowest TPRs among the three methods. Increasing the sample size *n* or decreasing the number of exposures *m* could achieve a higher TPR. AMC-based g-computation greatly outperforms WQS and quantile-based g-computation when the effects are non-monotonic. In addition, the average False Positive Rates (FPRs) of AMC-based g-computation were the lowest in all scenarios under monotonic or non-monotonic effects (Supplementary Table 5).

**Table 1:** Average True Positive Rates (TPRs) of the three methods under monotonic effects. Average TPR was calculated across 1000 simulations; *m* was the number of exposures; *n* was the sample size.

	m = 30			m = 50		
	n = 500	n = 1000	n = 1500	n = 500	n = 1000	n = 1500
AMC-based g-computation	72.2%	87.9%	94.8%	63.7%	82.0%	92.1%
Quantile-based g-computation	69.9%	85.9%	93.3%	60.9%	79.6%	89.6%
WQS	51.1%	65.2%	73.6%	39.3%	54.5%	64.4%

**Table 2:** Average True Positive Rates (TPRs) of the three methods under non-monotonic effects. Average TPR was calculated across 1000 simulations; *m* was the number of exposures; *n* was the sample size.

	m = 10			m = 20		
	n = 500	n = 1000	n = 1500	n = 500	n = 1000	n = 1500
AMC-based g-computation	78.0%	84.1%	86.6%	72.8%	82.6%	86.0%
Quantile-based g-computation	16.6%	14.4%	11.9%	15.4%	14.7%	12.5%
WQS	17.8%	17.0%	15.2%	15.8%	17.0%	15.4%

### Application to toenail trace element mixture data from the New Hampshire Bladder Cancer Study

We applied AMC-based g-computation to identify the associations between the mixture of 12 toenail trace elements and the risk of non-muscle invasive bladder cancer (NMIBC). As shown in Table 3, NMIBC cases and controls were similar with regard to the matching factors (age and gender). Most cases and controls were men, consistent with what is known about the gender-specific incidence of bladder cancer^17–19^. Cases were more likely to be smokers (including former and current smokers), less likely to complete a higher education, and more likely to have high-risk occupations than controls. The mean and median of toenail trace element concentrations for cases and controls are provided in Supplementary Table 1.

**Table 3:** Demographics of participants, New Hampshire Bladder Cancer Study, 2002–2004^a^.

	NMIBC cases (N = 265)	Controls (N = 353)
**Age**		
Mean (SD)	65.1 (10.3)	64.6 (10.6)
**Gender**		
Male	193 (72.8%)	253 (71.7%)
Female	72 (27.2%)	100 (28.3%)
**Smoking**		
Non-smoker	41 (15.5%)	137 (38.8%)
Former smoker^b^	144 (54.3%)	165 (46.7%)
Current Smoker^c^	80 (30.2%)	51 (14.4%)
**Education**		
High School	120 (45.3%)	132 (37.4%)
College	107 (40.4%)	155 (43.9%)
Postgraduate	38 (14.3%)	66 (18.7%)
**Occupation**		
High Risk	133 (50.2%)	112 (31.7%)
Low Risk	132 (49.8%)	241 (68.3%)

^a^Participants were 265 non-muscle invasive bladder cancer (NMIBC) cases and 353 controls of the New Hampshire Bladder Cancer Study.

^b^“Former smokers” quit smoking more than 1 year before they were diagnosed as cases or selected as controls.

^c^“Current smokers” still smoked or quit smoking less than 1 year before they were diagnosed as cases or selected as controls.

For consistent interpretation, we set the number of categorizations to be 3 so that each element was grouped into high, medium, and low levels by AMC. AMC-based g-computation was applied to log-transformed toenail trace element concentrations. Because the distributions of the toenail trace element exposures were right-skewed even after log-transformation, there is a possibility that only a few extreme large values may be grouped into the high-level category, leading to reduced statistical power. For this analysis, we required that no category would be assigned less than 10% of the total samples size among cases and controls combined. When applying g-computation, we used dummy variables to allow for a nonlinear exposure-outcome relationship. The cut points and sample sizes in each exposure level are shown in Supplementary Table 2. We bootstrapped the original dataset 1000 times and repeated g-computation to estimate a 95% confidence interval (CI).

**Figure 2:**
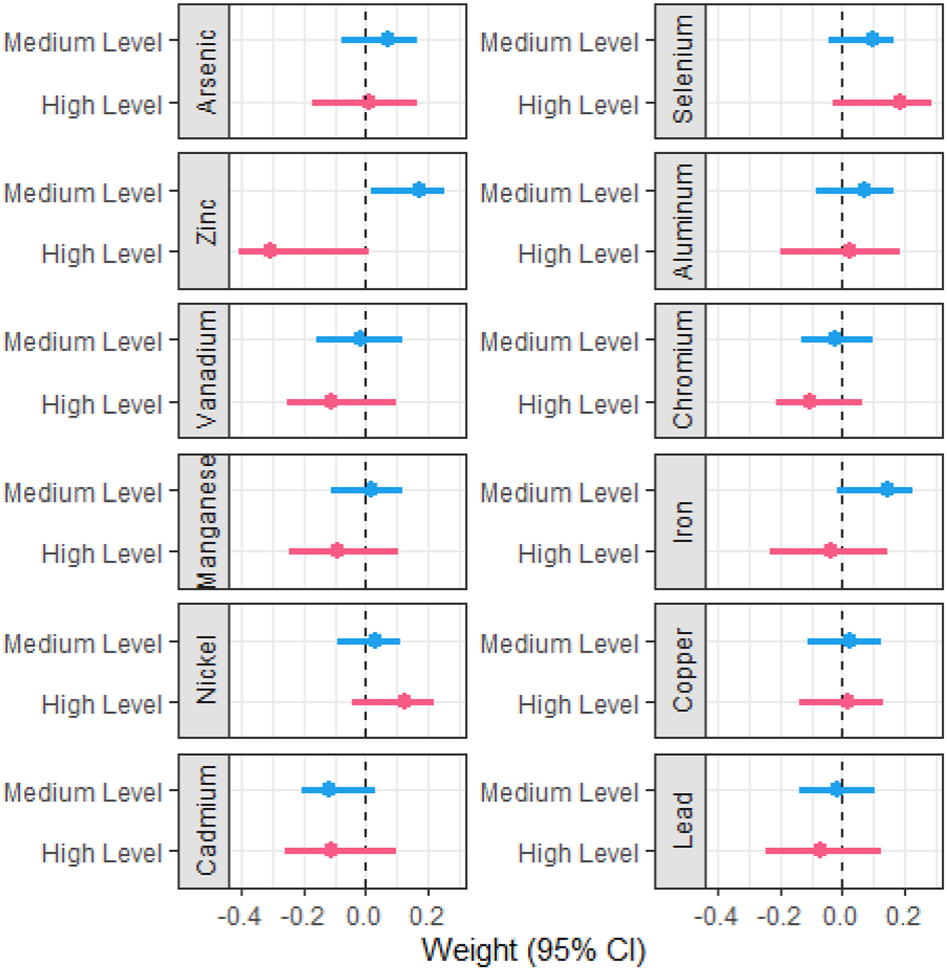
Weights and 95% confidence intervals of toenail trace element exposures in the AMC-based g-computation model adjusted for age, gender, smoking, education, and high-risk occupation. Low, medium, and high levels were categorized by AMC. Trace elements were transformed to dummy variables with the low-level set as the reference category. Weights were calculated by g-computation using R package “qgcomp”. 95% CIs were calculated by bootstrapping the original dataset 1000 times and performing repeated g-computation. All models adjusted for age, gender, smoking, education, and high-risk occupation. Positive weights and negative weights together sum to one.

The weights for each level of exposures from AMC-based g-computation with covariate adjustment and their 95% CIs are shown in Fig. 2 and Supplementary Table 3. The reference category for each element was the low-level category. The weights for the medium-level and high-level categories of each element were observed to differ in magnitude, but most were in the same direction. Toenail arsenic, selenium, aluminum, iron, nickel, and copper had positive weights in both medium- and high-level categories, while vanadium, manganese, cadmium, and lead had negative weights in both medium- and high-level categories. The medium-level vs. low-level of toenail zinc had a statistically significant positive association with bladder cancer risk (w = 0.172; 95% CI 0.016 to 0.254). No statistically significant associations were identified for other trace elements. The joint mixture effect (ψ = 0.633; 95% CI − 1.40 to 1.04; equivalent to the odds ratio OR = 1.88; 95% CI 0.25 to 2.83) suggested no statistically significant overall association between the toenail trace element mixture and risk of NMIBC.

## Discussion

We proposed AMC-based g-computation to provide a more flexible categorization approach than quantile-based g-computation and WQS. Our simulation study demonstrates that AMC-based g-computation reduces bias in coefficient estimation and improves accuracy in variable selection. Our proposed method uses empirical thresholds to categorize the data and maximize the between-group variance, thus minimizing information loss. AMC-based g-computation performed better than quantile-based g-computation and WQS at estimating the joint mixture effect by offering flexible, data-adaptable thresholds for categorization while still accounting for direction of effects. When the directional homogeneity assumption does not hold, both AMC-based and quantile-based g-computation greatly outperforms WQS, which does not account for direction of effects. We also found that our method outperformed the other two methods in variable selection. A potential drawback of our method is that the number of categories *k* may not be the global convex minimizing the p-value of F-test. In its current implementation, we restrict $k \le 10$ to limit the computational burden. K-means can be a viable alternative to linear search categorization. AMC procedure has a built-in procedure to select the value of *k* based on the p-value of F-test. However, in real data analysis, when exposures are categorized into different number of groups it may be difficult to interpret the finding. Therefore, we fixed *k* to be 3 in the analysis of trace elements and bladder cancer risk.

The association between trace element concentrations and the health outcomes can be complex and nonlinear. The human body needs essential trace elements to maintain cellular functions, but excess levels can be toxic^20–22^. In addition, associations can be stronger in a certain range of concentration but weaker outside that range. For example, a study reported toenail arsenic exposure has an inverse association with BMI comparing the 3rd quartile vs. the 1st quartile, but not for the 4th quartile^23^. Therefore, we applied AMC-based g-computation to detect a possible nonlinear relationship between exposure and outcome.

Previous studies have reported on the association between toenail elements and bladder cancer risk^24,25^. A study in Finland^25^ reported no association between toenail selenium concentration and bladder cancer. In contrast to our findings, a Netherlands Cohort Study^24^ identified an inverse association between toenail selenium and bladder cancer risk. A possible reason for the inconsistency with our findings is the very different overall distribution of selenium. In the Netherlands study, the 1st quintile of toenail selenium concentration was ≤ 0.483 μg/g and the 5th quintile was > 0.630 μg/g. However, in the New Hampshire Bladder Cancer Study toenail selenium concentration was much higher, the 1st quintile was ≤ 0.781 μg/g and 5th quintile was > 1.036 μg/g. In fact, this difference in distribution makes the results incomparable given that the selenium concentration in the 1st quintile (reference level) of the New Hampshire study was higher than the 5th quintile of the Netherlands study. The Netherlands study reported that their covariate-adjusted incidence rate ratios (RR) were 1.09, 0.55, 0.63 and 0.67 respectively for 2nd–5th quintiles vs. 1st quintile, with 0.55, 0.63 and 0.67 being statistically significant, suggesting an inverse, but possibly non-linear association. Other reasons for the inconsistency could be the different study periods and geographic locations, different measurement techniques, measurement errors, statistical methods, and choices for covariate adjustment.

Our mixture analysis found no statistically significant association between the toenail trace elements as a mixture and bladder cancer risk, but did suggest higher zinc may be associated with an increased risk. The weight for medium-level zinc (116.7–145.5 μg/g) vs. low-level zinc (39.5–116.2 μg/g) was positively associated with bladder cancer risk, which is consistent with previous studies of urinary zinc^20,26,27^, but in the opposite direction of previous studies of serum zinc^26,28,29^. Previous studies have observed that high arsenic concentration in drinking water is associated with higher bladder cancer risk^15,30,31^, yet studies of toenail arsenic have reported no association with bladder cancer risk^32^ or associations only in subgroups such as smokers^33^. Different substrates could also lead to varying results. Longnecker et al. observed that the Pearson correlation between dietary selenium intake and biomarkers in descending order were urine, whole blood, serum, and toenail^34^.

Relaxing the requirement for directional homogeneity enables assessment of more diverse mixtures. Different components of a mixture can have different directions of effect on a health outcome. For example, total cholesterol in blood is a mixture, with high low-density lipoprotein (LDL) cholesterol associated with increased risk of coronary heart disease (CHD) risk and high high-density lipoprotein (HDL) cholesterol associated with decreased CHD risk^35–37^. Furthermore, each exposure within a mixture can have non-linear relationship with the outcome. We used dummy variables to allow for a nonlinear exposure-outcome relationship in our real data analysis. We observed an inverted U-shaped relationship between toenail zinc concentrations and risk of non-muscle invasive bladder cancer: relative to low-level zinc, medium-level zinc had a statistically significant positive association (β = 0.17; 95% CI 0.02 to 0.25), but an inverse association that was not significant for high-level zinc (β = − 0.30; 95% CI − 0.41 to 0.01). A biological mechanism for this inverted U-shaped relationship is unclear.

Further studies are required to confirm this finding and clarify a functional role of zinc in bladder cancer development. Among the study limitations, longitudinal pre-diagnostic concentrations of trace elements were not available, and we cannot exclude the possibility that observed exposure levels were caused by the disease itself or post-diagnosis treatment. A cohort study or nested case–control study design would help ensure that trace element exposures were measured prior to diagnosis or the onset of preclinical disease^38,39^.

In summary, we proposed an adaptation to the method of quantile-based g-computation that categorizes exposures based on the observed within and between category variance to improve mixture effect estimation performance and variable selection. We applied AMC-based g-computation to toenail trace element data from the New Hampshire Bladder Cancer Study, and it can be applied to investigate the association between mixtures and other diseases, such as lung cancer, breast cancer and neurodegenerative diseases. This method may help unveil the complex relationship between mixture and health outcomes.

## Methods and materials

### Loss function for categorization

Without loss of generality, suppose that there are $n$ observations for each exposure. For a given category number $k \ge 2$, let $f\left( {n,\;k} \right)$ denote the categorization which classifies $n$ observations of an exposure variable into $k$ categories $\left( {C_{1} , \ldots ,C_{k} } \right)$. Let $C_{1} = \left\{ {X_{11} ,\;X_{12} , \ldots ,X_{{1n_{1} }} } \right\}$, $C_{2} = \left\{ {X_{21} ,{ }\;X_{22} , \ldots ,X_{{2n_{2} }} } \right\}$,…, $C_{k} = \left\{ {X_{{{\text{k}}1}} ,\;X_{{{\text{k}}2}} , \ldots ,X_{{kn_{k} }} } \right\}$, where $n_{1} + n_{2} \cdots + n_{{\text{k}}} = n.{ }$ The goal is to identify the categorization rule $f\left( {n,\;k} \right)$ that minimizes the within-category variation and maximizes the between-category variation. Here, we propose to employ the F-statistic^40,41^ as the criterion function and define the loss function as the reciprocal of the F-statistic:

$$L\left( {f\left( {n,k} \right)} \right) = \frac{{Variation_{within} /\left( {n - k} \right)}}{{Variation_{between} /\left( {k - 1} \right)}}$$

where $Variation_{within} = \mathop \sum \nolimits_{i = 1}^{k} \mathop \sum \nolimits_{j = 1}^{{n_{i} }} \left( {X_{ij} - \overline{X}_{i} } \right)^{2} ,$ denoting the within-category variation; $Variation_{between} = \mathop \sum \nolimits_{i = 1}^{k} n_{i} \left( {\overline{X}_{i} - \overline{X}} \right)^{2}$, denoting the between-category variation; the mean of the *i*th category is denoted by $\overline{X}_{i}$, and the mean over all categories is denoted by $\overline{X}$. We can get the optimal categorization by minimizing this loss function. Note that optimal categorization refers to exposure variation and not necessarily optimal estimation of the mixture effect.

### Increase categories by linear search

When categorizing the *n* observations into *k* categories an exhaustive search identifying all possible classifications can lead to computational complexity given by the combination formula:

$$\left( {\begin{array}{*{20}c} {n - 1} \\ {k - 1} \\ \end{array} } \right) = \frac{{\left( {n - 1} \right)\left( {n - 2} \right) \cdots \left( {n - k + 1} \right)}}{{\left( {k - 1} \right)\left( {k - 2} \right) \cdots 1}}$$

The computational complexity will become extremely large when the sample size *n* is large. To apply this method in an efficient way, we increase *k* by linear search, which has lower computational complexity, $k\left( {n - 1} \right),$ compared to the exhaustive search. In this way, the algorithm will first set $k = 2$ for a dichotomous categorization. Fixing this initial boundary, we will continue to find another boundary for $k = 3$, and so on. In general, the *k*th category will be defined based on fixing the previous $k - 1$ categories. As the number of categories *k* is unknown, we can select an appropriate *k* that minimizes the p-value from the F test. The degree of freedom of the F test will be used to account for the number of categories *k*. After we determined the optimal thresholds using AMC, we implement g-computation to estimate the joint mixture effect using the R package ‘qgcomp’^10^.

**Figure 3:**
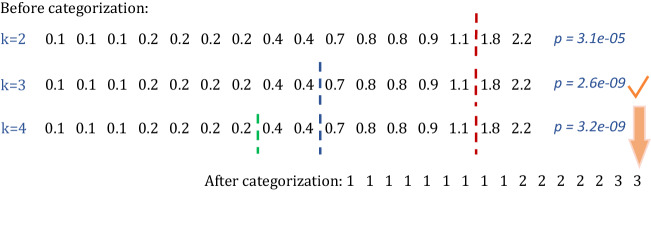
Overview of AMC procedure.

To demonstrate the steps of our method, consider an example of categorizing a variable with 16 observations as displayed in Fig. 3:

1. Starting with $k = 2$, we sort the data, exhaustively divide the sorted data into two categories, and calculate the loss function. The optimal categorization is the one with the smallest loss function.

2. Increase *k* by 1. Keep the cutoff from previous step(s) and search for another cutoff that minimizes the loss function.

3. Repeat the previous step until the increase of *k* does not result in a p-value for the F test that continues to decrease. The *k* that minimizes the p-value of F test will be our final choice. We do not consider $k > 10$ to limit the computational burden.

In Fig. 3, the p-value of F-test decreases when increasing *k* from 2 to 3, but it increases after increasing *k* to 4, so AMC will divide this example into 3 categories. The above steps are used to categorize exposure variables, where F-test is applied to a single exposure each time and then applied sequentially to every exposure. Next, g-computation is employed to estimate the mixture effect.

### Simulation

We performed two simulations to evaluate the bias in estimating the overall mixture effect and the accuracy to identify the true predictors.

In the first simulation, we evaluated the performance of the three methods (AMC-based g-computation, quantile-based g-computation, and WQS) when the number of exposures and sample size varies. We simulated data with a total of *m* continuous exposures each with *n* sample size and set *m* equal to 5, and *n* equal to 200. Let $Z_{j} \left( {1 \le j \le m} \right)$ represent the latent-state factor corresponding to the *j*th exposure $X_{j}$, and each exposure has 4 hidden states as follows:

$$\left( {Z_{j0} ,\;Z_{j1} ,\;Z_{j2} ,\;Z_{j3} } \right) = \left( {0,{ }\;1,{ }\;2,{ }\;3} \right)$$

Assume the probability of each hidden state is $\alpha = \left( {{\upalpha }_{j0} ,\;{\upalpha }_{j1} ,\;{\upalpha }_{j2} ,\;{\upalpha }_{j3} } \right),$ where $0 < {\upalpha }_{j0} ,\;{\upalpha }_{j1} ,\;{\upalpha }_{j2} ,\;{\upalpha }_{j3} < 1,$ and ${\upalpha }_{j0} + {\upalpha }_{j1} + {\upalpha }_{j2} + {\upalpha }_{j3} = 1.$ $Z_{j}$ is generated from a multinomial distribution with parameters *n* and $\alpha$. The value of $X_{j}$ is simulated from a mixture of normal distributions $f_{j0} ,{ }\;f_{j1} ,\;{ }f_{j2} ,\;f_{j3}$ corresponding to the normal distribution at each hidden state, $X_{j} \sim \alpha_{j0} f_{j0} + \alpha_{j1} f_{j1} + \alpha_{j2} f_{j2} + \alpha_{j3} f_{j3}$.

Among the *m* simulated exposures, assume only $X_{1}$, $X_{2}$ have effect on the outcome while $X_{3} , \ldots , X_{m}$ do not. The outcome *Y* is generated by the latent states $Z_{1}$ and $Z_{2}$ using the formula below:

$$Y = \beta_{1} Z_{1} + \beta_{2} Z_{2} + \varepsilon$$

where $\varepsilon \sim N\left( {0,\;1} \right)$. The joint mixture effect is defined as $\psi = \beta_{1} + \beta_{2}$.

We compared the bias of the estimated joint mixture effect among the three methods, where this bias is defined as $\psi_{estimate} - \psi_{true}$. The simulation was repeated 1000 times to calculate the average bias for each method. Parameters were set to be $f_{j0} = N\left( {1, \;0.33^{2} } \right),$ $f_{j1} = N\left( {2, \;0.33^{2} } \right), \;f_{j2} = N\left( {3, \;0.33^{2} } \right), \; f_{j3} = N\left( {4, \;0.33^{2} } \right)$. We used equal proportions for the four distributions and set the parameters to be ${\upalpha }_{j0} = {\upalpha }_{j1} = {\upalpha }_{j2} = {\upalpha }_{j3} = 0.25$ (Scenarios 1 and 2 in Fig. 1). We also used unequal proportions with ${\upalpha }_{j0} = 0.4,{ }\;{\upalpha }_{j1} = 0.3,{ }\;{\upalpha }_{j2} = 0.2,{ }\;{\upalpha }_{j3} = 0.1$ (Scenarios 3 and 4). As for directions of exposure effects, we considered two scenarios: (1) Effects are both positive, set $\beta_{1} = \beta_{2} = 0.5$; $\psi_{true} = 1$; (2) Effects are positive and negative, set $\beta_{1} = 1.5,\; \beta_{2} = - 0.5$; $\psi_{true} = 1$.

In the second simulation, parameters were set to be ${\upalpha }_{j0} = 0.4,\;{{ \upalpha }}_{j1} = 0.3,{ }\;{\upalpha }_{j2} = 0.2,{ }\;{\upalpha }_{j3} = 0.1;$ $f_{j0} = N\left( {1, 0.33^{2} } \right),$ $f_{j1} = N\left( {2, 0.33^{2} } \right), \;f_{j2} = N\left( {3, 0.33^{2} } \right), \;f_{j3} = N\left( {4, 0.33^{2} } \right)$, and sample size *n* equals to 500, 1000 or 1500. First, in a simulation of monotonic exposure effects, we assumed the outcome *Y* was associated with the first 10 exposures and was defined by the linear sum of their latent states:

$$Y = \mathop \sum \limits_{i = 1}^{10} \beta_{i} Z_{i} + \varepsilon$$

where $\varepsilon \sim N\left( {0,\;1} \right), \; \beta_{1} = \beta_{2} = \beta_{3} = \beta_{4} = \beta_{5} = \beta_{6} = \beta_{7} = 0.1,$ and $\beta_{8} = \beta_{9} = \beta_{10} = - 0.1.$ The true joint exposure effect $\psi_{true}$ equals 0.4 by definition^10^. We set the total number of exposures *m* equal to 30 or 50. We applied the three methods on the simulated data to calculate the exposure weights for $X_{1} , \;X_{2} , \ldots , X_{m} .$ The weights were sorted in descending order. For the positive weights, we counted the number of *X*_1_–*X*_7_ if they were among the largest 7 weights and denoted this count as *TP*_*1*_. For the negative weights, we counted the number of *X*_8_–*X*_10_ if they were among the smallest 3 weights and denoted this count as *TP*_*2*_. We used true positive rate (TPR) as the criterion of variable-selection performance, defined as $TPR = TP/P$. Here, $TP = TP_{1} + TP_{2}$, represents the number of correctly-identified variables and *P* equals 10, representing the number of true exposures.

Second, we simulated non-monotonic effect of exposures. Each latent-state factor $Z_{j} \left( {1 \le j \le m} \right)$ were transformed to a set of four dummy variables $Z_{j}^{dummy} = \left( {Z_{j0}^{dummy} ,{ }\;Z_{j1}^{dummy} ,\;{ }Z_{j2}^{dummy} ,\;Z_{j3}^{dummy} } \right).$ The corresponding coefficient vector was $\beta_{j} = \left( {\beta_{j0} ,\;\beta_{j1} ,\;\beta_{j2} ,\;\beta_{j3} } \right) = \left( {0,{ }\;1,{ }\; - 1,{ }\;0} \right)$, including positive, negative and zero values representing non-monotonic effects. We assumed the outcome *Y* was associated with the first 5 exposures:

$$Y = \mathop \sum \limits_{j = 1}^{5} { }Z_{j}^{dummy} \beta_{j}^{T} + \varepsilon$$

where $\varepsilon \sim N\left( {0,\;1} \right)$. We set *m* equal to 10 or 20; ${\upalpha }_{j0} = 0.4,{ }\;{\upalpha }_{j1} = 0.3,\;{\upalpha }_{j2} = 0.2,\;{\upalpha }_{j3} = 0.1;$ $f_{j0} = N\left( {1, \;0.33^{2} } \right),$ $f_{j1} = N\left( {2, \;0.33^{2} } \right), \;f_{j2} = N\left( {3, \;0.33^{2} } \right),\; f_{j3} = N\left( {4, \;0.33^{2} } \right)$, and sample size *n* equal to 500, 1000 or 1500. We followed the same procedure described above to calculate the TPR. For the positive weights, we counted the number of $X_{j1 } \left( {j = 1, \;2,\; 3, \;4,\; 5} \right)$ if they were among the largest 5 weights and denoted this count as *TP*_*1*_. For the negative weights, we counted the number of $X_{j2 } \left( {j = 1, \;2, \;3,\; 4, \;5} \right)$ if they were among the smallest 5 weights and denoted this count as *TP*_*2*_. TPR is defined as $TPR = \left( {TP_{1} + TP_{2} } \right)/10$ to compare the variable-selection performance.

### Dataset and pre-processing for the application of real data

Participants of the New Hampshire Bladder Cancer Study^42^ used in this analysis were recruited from January 1, 2002 to July 31, 2004. New Hampshire Bladder Cancer Study is a population-based case–control study conducted among New Hampshire residents^43^. The cases were 396 patients diagnosed with histologically-confirmed urothelial bladder cancer at ages 31–79, identified through the New Hampshire State Cancer Registry^44^. A total of 426 controls, frequency-matched to cases by gender and age, were selected from the state Department of Motor Vehicles (DMV) records and the Centers for Medicare and Medicaid Services (CMS) beneficiary records^45^. The New Hampshire Bladder Cancer Study was conducted in accordance with the guidelines and regulations of the Declaration of Helsinki. The study was approved by the Committee for the Protection of Human Subjects at Dartmouth College, and all participants provided informed consent.

Participants of the New Hampshire Bladder Cancer Study provided toenail clippings for quantification of trace element concentrations^46–48^. Inductively coupled plasma mass spectrometry (ICP-MS)^46^ was used to measure the concentrations of 12 trace elements (arsenic, selenium, zinc, aluminum, vanadium, chromium, manganese, iron, nickel, copper, cadmium, and lead). Analysis of the toenail samples was conducted at the Dartmouth Trace Element Analysis Core, which has analyzed over 10,000 nail samples, employs a rigorous quality control program, and has participated in the external QMEQAS proficiency program operated by the Centre for Toxicology, Quebec since 2011.

We restricted our analysis to 327 cases with non-muscle invasive bladder cancer (NMIBC) because NMIBC comprised 83% of the bladder cancer cases in the study. Among the NMIBC cases and controls, 55 cases and 59 controls did not provide toenail samples. Another 7 cases and 14 controls were excluded due to missing demographic information or missing concentrations of trace elements. In total, 265 cases and 353 controls were included in logistic regression modeling. The model covariates included age, gender, smoking status, education, and high-risk occupation. High-risk occupation included vehicle repairers, electrical and electronics repairers, precision metalworkers, military occupations, drafting occupations and others as described by Colt et al.^49^. Trace element concentrations below the limit of detection (LOD) were imputed using the LOD divided by 2^50,51^.

## Supplementary Information

Supplementary Tables.

## Data Availability

De-identified data described in the manuscript will be made available upon reasonable request pending approval of an application for data use addressed to Dr. Margaret Karagas (Margaret.R.Karagas@dartmouth.edu), and execution of a data use agreement or material transfer agreement with Dartmouth College. R code implementing our proposed method can be accessed at https://github.com/sitingLi/amc. All simulation code is available at https://github.com/sitingLi/AMC_simulations.
