# Supplementary material for: Adaptive-mixture-categorization (AMC)-based g-computation and its application to trace element mixtures and bladder cancer risk
Source: Sci Rep. 2022 Oct 25;12:17841. doi: 10.1038/s41598-022-21747-7 (PMC9596719; doi:10.1038/s41598-022-21747-7)
Supplement: Supplementary file 1 — Supplementary Tables. [file 41598_2022_21747_MOESM1_ESM.docx]

Supplementary Information

**Supplementary Table 1.** Descriptive Statistics of toenail trace element concentrations among 265 non-muscle invasive bladder cancer (NMIBC) cases and 353 controls, New Hampshire, 2002-2004^a^. Mean, median, minimum, maximum, and standard error of trace elements are listed below for NMIBC cases and controls separately.

|  | **NMIBC Cases (N=265)** | **Controls (N=353)** |
| --- | --- | --- |
| **Arsenic (μg/g)** |  |  |
| Mean (SD) | 0.085 (0.110) | 0.091 (0.107) |
| Median [Min, Max] | 0.059 [0.021, 1.48] | 0.062 [0.018, 1.28] |
| **Selenium (μg/g)** |  |  |
| Mean (SD) | 0.930 (0.223) | 0.944 (0.476) |
| Median [Min, Max] | 0.892 [0.620, 2.50] | 0.886 [0.458, 8.99] |
| **Zinc (μg/g)** |  |  |
| Mean (SD) | 113 (26.9) | 116 (47.7) |
| Median [Min, Max] | 110 [52.4, 341] | 108 [39.5, 766] |
| **Aluminum (μg/g)** |  |  |
| Mean (SD) | 18.0 (45.2) | 16.4 (21.6) |
| Median [Min, Max] | 9.02 [1.52, 612] | 10.1 [1.47, 259] |
| **Vanadium (μg/g)** |  |  |
| Mean (SD) | 0.020 (0.028) | 0.022 (0.029) |
| Median [Min, Max] | 0.011 [0.0002, 0.341] | 0.014 [0.0002, 0.300] |
| **Chromium (μg/g)** |  |  |
| Mean (SD) | 0.732 (1.59) | 0.952 (4.31) |
| Median [Min, Max] | 0.293 [0.013, 13.2] | 0.376 [0.010, 79.2] |
| **Manganese (μg/g)** |  |  |
| Mean (SD) | 0.718 (1.13) | 0.890 (1.58) |
| Median [Min, Max] | 0.361 [0.040, 7.83] | 0.430 [0.066, 18.6] |
| **Iron (μg/g)** |  |  |
| Mean (SD) | 19.7 (20.8) | 23.7 (25.6) |
| Median [Min, Max] | 13.5 [4.82, 248] | 15.7 [3.71, 214] |
| **Nickel (μg/g)** |  |  |
| Mean (SD) | 4.09 (20.0) | 3.07 (11.9) |
| Median [Min, Max] | 0.500 [0.003, 246] | 0.469 [0.003, 111] |
| **Copper (μg/g)** |  |  |
| Mean (SD) | 4.71 (4.15) | 4.85 (4.45) |
| Median [Min, Max] | 3.69 [1.66, 41.5] | 3.77 [0.747, 50.9] |
| **Cadmium (μg/g)** |  |  |
| Mean (SD) | 0.030 (0.044) | 0.034 (0.051) |
| Median [Min, Max] | 0.016 [0.001, 0.429] | 0.020 [0.001, 0.455] |
| **Lead (μg/g)** |  |  |
| Mean (SD) | 0.653 (4.44) | 0.674 (3.41) |
| Median [Min, Max] | 0.196 [0.020, 71.9] | 0.216 [0.030, 61.7] |

SD = Standard deviation; Min = Minimum; Max = Maximum

^a^ Participants were from the New Hampshire Bladder Cancer Study. Concentrations were measure by inductively coupled plasma mass spectrometry (ICP-MS).

**Supplementary Table 2.** Sample sizes and cutoff points of toenail trace element levels. Levels are categorized by AMC-based g-computation.

| **Exposure** | **Low Level** | | **Medium Level** | | **High Level** | |
| --- | --- | --- | --- | --- | --- | --- |
|  | **Sample Size** | **Concentration**  **(μg/g)^a^** | **Sample Size** | **Concentration**  **(μg/g)** | **Sample Size** | **Concentration**  **(μg/g)** |
| Arsenic | 437 | 0.018, 0.084 | 119 | 0.085, 0.160 | 62 | 0.161, 1.481 |
| Selenium | 397 | 0.458, 0.950 | 159 | 0.952, 1.143 | 62 | 1.145, 8.987 |
| Zinc | 389 | 39.510, 116.245 | 167 | 116.715, 145.500 | 62 | 145.842, 766.257 |
| Aluminum | 352 | 1.465, 11.354 | 204 | 11.570, 31.432 | 62 | 32.111, 611.936 |
| Vanadium | 69 | 0.0002, 0.004 | 229 | 0.004, 0.012 | 320 | 0.012, 0.341 |
| Chromium | 327 | 0.010, 0.366 | 192 | 0.375, 1.158 | 99 | 1.204, 79.199 |
| Manganese | 360 | 0.040, 0.511 | 189 | 0.518, 1.552 | 69 | 1.579, 18.550 |
| Iron | 211 | 3.714, 11.252 | 222 | 11.365, 20.870 | 185 | 21.030, 247.830 |
| Nickel | 243 | 0.003, 0.332 | 297 | 0.339, 2.986 | 78 | 3.258, 246.039 |
| Copper | 204 | 0.747, 3.218 | 284 | 3.237, 5.302 | 130 | 5.321, 50.897 |
| Cadmium | 322 | 0.001, 0.019 | 227 | 0.019, 0.059 | 69 | 0.060, 0.455 |
| Lead | 379 | 0.020, 0.283 | 177 | 0.285, 0.850 | 62 | 0.896, 71.863 |

^a^ Showing the lowest concentration and the highest concentration within each level. Rounding to 3 decimal places.

**Supplementary Table 3.** Weights and 95% confidence intervals (CIs) of medium-level and high-level trace element exposures^a^

| **Exposure** | **Medium-Level Weight (95% CI)** | **High-Level Weight (95% CI)** |
| --- | --- | --- |
| Arsenic | 0.074 (-0.080, 0.167) | 0.013 (-0.173, 0.164) |
| Selenium | 0.098 (-0.043, 0.168) | 0.185 (-0.027, 0.287) |
| Zinc | 0.172 (0.016, 0.254) | -0.304 (-0.408, 0.009) |
| Aluminum | 0.070 (-0.086, 0.165) | 0.024 (-0.196, 0.183) |
| Vanadium | -0.016 (-0.159, 0.121) | -0.114 (-0.251, 0.099) |
| Chromium | -0.021 (-0.129, 0.097) | -0.105 (-0.215, 0.066) |
| Manganese | 0.018 (-0.114, 0.121) | -0.089 (-0.246, 0.103) |
| Iron | 0.147 (-0.018, 0.225) | -0.034 (-0.231, 0.142) |
| Nickel | 0.029 (-0.088, 0.112) | 0.126 (-0.042, 0.217) |
| Copper | 0.026 (-0.110, 0.122) | 0.017 (-0.136, 0.132) |
| Cadmium | -0.118 (-0.207, 0.030) | -0.110 (-0.261, 0.097) |
| Lead | -0.018 (-0.138, 0.106) | -0.071 (-0.243, 0.126) |

^a^ Low, medium, and high categories were categorized by AMC. Trace elements were transformed to dummy variables and low-level was the reference category of each trace element. Weights were calculated by g-computation using R package “qgcomp”. 95% CIs were calculated by bootstrapping the original dataset 1000 times and repeated g-computation. Age, gender, smoking, education, and occupation were adjusted in the model.

**Supplementary Table 4.** Weights and 95% confidence intervals (CIs) of medium-level and high-level trace element exposures^a^ by smoking status

| **Exposure** | **Medium-Level Weight (95% CI)** | **High-Level Weight (95% CI)** |
| --- | --- | --- |
| Arsenic | 0.029 (-0.097, 0.137) | 0.030 (-0.224, 0.168) |
| Selenium | 0.140 (-0.035, 0.227) | 0.179 (-0.016, 0.255) |
| Zinc | 0.053 (-0.081, 0.193) | -0.067 (-0.198, 0.085) |
| Aluminum | -0.140 (-0.231, 0.033) | -0.023 (-0.187, 0.179) |
| Vanadium | -0.153 (-0.228, 0.029) | -0.019 (-0.184, 0.144) |
| Chromium | 0.008 (-0.130, 0.130) | 0.043 (-0.155, 0.206) |
| Manganese | 0.064 (-0.070, 0.171) | -0.227 (-0.411, 0.020) |
| Iron | -0.047 (-0.162, 0.089) | -0.181 (-0.358, 0.039) |
| Nickel | 0.077 (-0.053, 0.163) | 0.168 (-0.029, 0.262) |
| Copper | 0.021 (-0.090, 0.123) | -0.004 (-0.160, 0.145) |
| Cadmium | -0.052 (-0.144, 0.076) | -0.075 (-0.269, 0.109) |
| Lead | -0.011 (-0.155, 0.105) | 0.188 (-0.010, 0.284) |

**Supplementary Table 4-1.** Weights and 95% CIs of trace element exposures^a^ among non-smokers (41 cases and 137 controls)

| **Exposure** | **Medium-Level Weight (95% CI)** | **High-Level Weight (95% CI)** |
| --- | --- | --- |
| Arsenic | 0.029 (-0.100, 0.145) | 0.043 (-0.111, 0.167) |
| Selenium | 0.037 (-0.109, 0.153) | 0.158 (-0.063, 0.266) |
| Zinc | 0.272 (0.057, 0.313) | -0.010 (-0.205, 0.193) |
| Aluminum | 0.180 (-0.022, 0.264) | 0.087 (-0.173, 0.255) |
| Vanadium | 0.071 (-0.094, 0.194) | -0.010 (-0.176, 0.174) |
| Chromium | -0.065 (-0.159, 0.077) | -0.166 (-0.254, 0.044) |
| Manganese | -0.015 (-0.134, 0.129) | -0.026 (-0.210, 0.183) |
| Iron | -0.094 (-0.215, 0.104) | -0.090 (-0.269, 0.169) |
| Nickel | 0.055 (-0.116, 0.172) | 0.068 (-0.114, 0.180) |
| Copper | -0.025 (-0.133, 0.113) | -0.006 (-0.149, 0.146) |
| Cadmium | -0.072 (-0.156, 0.071) | -0.088 (-0.255, 0.130) |
| Lead | -0.085 (-0.181, 0.074) | -0.247 (-0.340, 0.024) |

**Supplementary Table 4-2.** Weights and 95% CIs of trace element exposures^a^ among smokers^b^ (224 cases and 216 controls)

^a^ Low, medium, and high categories were categorized by AMC. Trace elements were transformed to dummy variables and low-level was the reference category of each trace element. Weights were calculated by g-computation using R package “qgcomp”. 95% CIs were calculated by bootstrapping the original dataset 1000 times and repeated g-computation. Age, gender, education, and occupation were adjusted in the model.

^b^ Smokers include former smokers and current smokers.

**Supplementary Table 5.** Average False Positive Rates (FPRs) of the three methods

|  | **m = 30** | | | **m = 50** | | |
| --- | --- | --- | --- | --- | --- | --- |
|  | **n = 500** | **n = 1000** | **n = 1500** | **n = 500** | **n = 1000** | **n = 1500** |
| **AMC-based g-computation** | 13.9% | 6.0% | 2.6% | 9.1% | 4.5% | 2.0% |
| **Quantile-based g-computation** | 15.0% | 7.1% | 3.3% | 9.8% | 5.1% | 2.6% |
| **WQS** | 23.9% | 17.3% | 13.2% | 15.0% | 11.3% | 8.9% |

**Supplementary Table 5-1.** **Average False Positive Rates (FPRs) of the three methods under monotonic effects.** Average FPR was calculated across 1,000 simulations; *m* was the number of exposures; *n* was the sample size.

|  | **m = 10** | | | **m = 20** | | |
| --- | --- | --- | --- | --- | --- | --- |
|  | **n = 500** | **n = 1000** | **n = 1500** | **n = 500** | **n = 1000** | **n = 1500** |
| **AMC-based g-computation** | 11.0% | 8.0% | 6.7% | 5.4% | 3.5% | 2.8% |
| **Quantile-based g-computation** | 25.7% | 25.1% | 25.0% | 10.9% | 10.1% | 10.0% |
| **WQS** | 26.4% | 25.1% | 25.0% | 11.6% | 10.2% | 10.0% |

**Supplementary Table 5-2. Average False Positive Rates (FPRs) of the three methods under non-monotonic effects.** Average FPR was calculated across 1,000 simulations; *m* was the number of exposures; *n* was the sample size.
